# Correlating Interictal Spikes with Sigma and Delta Dynamics during Non-Rapid-Eye-Movement-Sleep

**DOI:** 10.3389/fneur.2017.00288

**Published:** 2017-06-22

**Authors:** Frédéric Zubler, Annalisa Rubino, Giorgio Lo Russo, Kaspar Schindler, Lino Nobili

**Affiliations:** ^1^“C. Munari” Center for Epilepsy Surgery, Department of Neuroscience, Niguarda Hospital, Milan, Italy; ^2^Department of Neurology, Inselspital—Bern University Hospital, University of Bern, Bern, Switzerland

**Keywords:** sleep, sleep instability, epileptic spikes, sigma power, slow wave activity, focal cortical dysplasia, stereo-EEG

## Abstract

Interictal spikes (IS) are one of the major hallmarks of epilepsy. Understanding the factors promoting or suppressing IS would increase our comprehension of epilepsy and possibly open new avenues for therapy. Sleep strongly influences epileptic activity, and the modulatory effects of the different sleep stages on IS have been studied for decades. However, several aspects are still disputed, in particular the role of sleep spindles and slow waves in the activation of IS during Non-REM sleep. Here, we correlate the rate of IS with quantitative measures derived from stereo-EEG during one Non-REM cycle in 10 patients suffering from drug-resistant epilepsy due to type 2 focal cortical dysplasia. We show that the IS rate (ISR) is positively correlated with sigma power (a surrogate for sleep-spindle density) but negatively correlated with delta power (surrogate for slow wave activity). In addition, we present two new indices for quantifying the spatial and temporal instability of sleep. We found that both instability indices are correlated with a high ISR. The main contribution of this study is to confirm the suppressive effect of stable deep sleep on IS. This result might influence future guidelines for therapy of patients suffering from epilepsy and sleep disorders.

## Introduction

A better understanding of the mechanisms underlying the generation of interictal epileptic activity would improve our comprehension of epilepsy (1–3). Since sleep strongly influences the production of interictal epileptic activity, the modulatory effects of sleep stages have been the object of both experimental and clinical studies in the last few decades (4). A large majority of studies indicate that non-rapid-eye-movement (NREM)-sleep increases epileptic activity, whereas REM suppresses both interictal and ictal manifestations of epilepsy (5–11). In the last few years, the relation between sleep and epilepsy has been investigated using invasive recording in drug-resistant patients during pre-surgical evaluation. Intracranial EEG is more accurate for the investigation of the relationship between sleep and epilepsy. First, because it allows the detection of interictal spikes (IS) that are not visible on the scalp (12, 13); second, because intracranial EEG offers a better spatial resolution highlighting the local behavior of both epileptic and physiological sleep activity (2, 13–15); finally, because the signal-to-noise ratio is higher in invasive than in non-invasive EEG (16). Recently, using Stero-EEG, it has been demonstrated that the suppressive effect of REM sleep on interictal epileptic activity is mainly observed during the occurrence of ocular movements [phasic REM sleep; (10, 11)], a phase characterized by significant reduction of synchronization at a local and global scale (11). Synchronization has also been considered to be the basis of spike production during NREM sleep. The two main EEG indicators of NREM-synchronization are sleep spindles (mostly found in sleep stage NREM2; N2) and slow waves (mostly produced during sleep stage NREM 3; N3). *In vivo*, both patterns are generated, or at least coordinated, by the thalamocortical loop and are thought to contribute to cortical synchronization through repetitive volleys of thalamocortical excitation for the former (17, 18) and through widespread synchronous alternations between firing and silent periods of excitatory cortical cells (“up” and “down” states) for the latter (17–20). However, the relative importance of spindles and slow waves in interictal activity in humans has been assessed so far mainly in scalp EEG. The results of these studies, based on spectral analysis with the power in sigma (12–16 Hz) and delta (0.5–4 Hz) bands as indirect quantifiers of sleep spindles and slow waves, respectively, remain controversial. Indeed, the production of interictal epileptic spikes (IS) seems to follow either sigma or delta power, depending on the type of epilepsy. IS rate (ISR) was correlated with sigma power in childhood epilepsy syndromes such as epilepsy with centro-temporal spikes or continuous spike wave in sleep (7, 8, 21), whereas ISR followed delta power in adults with structural epilepsy (6, 21). Not only the total delta power but also its variation over time [rate of delta increase (6), “cyclic alternating pattern” (22)] has been shown to modulate ISR.

A few studies were conducted with invasive recording during NREM sleep—however without direct comparison of ISR with temporal dynamics of sigma and delta power. Using foramen ovale electrodes in patients with temporal lobe epilepsy, Clemens et al. (12) found that on the scalp the ISR was higher during N3 than N2, whereas in simultaneous intracranial recordings it was the opposite. More recently, Frauscher et al. (23) showed that the timing of interictal epileptic activity occurrence was influenced by the phase, amplitude, and topographic spread of single slow waves; however, this last study did not assess the ISR or the possible role of sleep spindles.

Here, we set out to describe the relation between ISR and EEG markers of NREM sleep in a population of patients suffering from pharmaco-resistant epilepsy due to Type 2 Focal cortical dysplasia (FCD2). Several reasons dictated the choice of this particular etiology. The first one is that FCD2 is a localized lesion (24), and affected patients usually have very few symptoms beside focal epileptic seizures (25), suggesting that the rest of the brain produces more physiological sleep activity. IS are numerous, allowing for robust correlations, and are often present in the seizure-onset zone (SOZ) (26). Finally, in FCD2, the epileptic activity is particularly sensitive to sleep stages (25, 27).

We first describe the time course of ISR within and outside of the SOZ during one NREM-cycle. The dynamics of ISR is then correlated with those of delta and sigma power. Furthermore, we propose two new indices based on relative delta power (RDP) to quantify temporal and spatial instability of NREM sleep. Finally, the relative importance of these four variables in the association with ISR is assessed with a two-step linear model.

## Materials and Methods

### Patients and Data Acquisition

This study was carried out in accordance with the recommendations of the Ethics Commission of the Ospedale Niguarda. All patients gave written informed consent for retrospective analysis of their data in accordance with the Declaration of Helsinki. Patients with drug-resistant epilepsy who underwent stereo-EEG recording in the “C. Munari” Center for Epilepsy Surgery between July 2011 and December 2015 were retrospectively screened. Inclusion criteria were (i) the presence of concomitant scalp EEG electrodes (usually Fz and Cz), Electrooculogram, and submental EMG electrodes allowing sleep scoring; (ii) epilepsy caused by an FCD 2—recognized based on histopathological findings; (iii) at least one EEG contact within or at the immediate border of the FCD recognized based on neuroradiological findings and/or on typical intracerebral EEG pattern; (iv) presence of IS both within and outside of the SOZ (see definition below); and (v) complete seizure freedom (Engel class 1A) after surgical operation with at least 1 year of follow-up, confirming the location of the SOZ.

Intracerebral EEGs were recorded by Microdeep intracerebral electrodes D08 (Dixi Medical) or Depth Electrodes Range 2069 (Alcis, France) comporting 5–18 contacts distant from 1.5 mm. EEG signals were acquired with a Neurofax EEG-1100 system (Nihon Koden, Tokyo, Japan), sampled at 1 kHz with 16-bit resolution. The recordings were imported into Matlab 2016a (MathWorks, Natick, MA, USA) and digitally down-sampled to 500 Hz (after low-pass filtering with an eighth order Chebyshev Type I filter with a cut-off frequency of 200 Hz) before analysis.

We considered only the contacts placed in the cortex [identified based on the co-registration of pre-implantation MRI and CT-scan post implantation, for details see Ref. (28)]. A bipolar montage without common contact between neighboring pairs was used. For justification of selecting a bipolar montage, see Figure S1 in Supplementary Material.

### Visual EEG Analysis

Sleep scoring was performed based on extracranial recordings on 30-s epochs according to the criteria of the American Academy of Sleep Medicine (29). For each patient, we analyzed the first NREM-cycle without epileptic seizures during which sleep stage N3 was reached. The beginning of the cycle was defined as the first N2 epoch not later followed by wakefulness; the end of the cycle was defined as the last epoch before occurrence of REM or wakefulness. To be able to compare the dynamics over NREM-cycles of different length, we divided each cycle into 30 non-overlapping segments. This number was chosen because it approximately corresponded to the duration in minutes of the shortest cycle.

The SOZ was identified based on the presence of low-amplitude fast activity at seizure onset in the intracranial recordings. Two bipolar stereo-EEG channels were selected for counting IS, one within and one outside the SOZ. The latter was chosen in the “diffusion zone” (DZ), namely in the brain region where the ictal discharge first spreads from the SOZ (11). IS were marked manually on both channels independently. The following operational criteria were used: a transient was marked as IS if it was (i) sharply configured and (ii) of high amplitude (at least three times the peak-to-peak amplitude of the background) and/or [disrupting the background and followed by a clear “wave”], and (iii) not being better described as another EEG transient such as a slow wave, K-complex, sleep spindle, or “brush.” A spike superimposed onto such a transient was not excluded if it fulfilled the criteria (i–iii). Polyspikes were counted as one instance of IS, whereby the spike with the highest amplitude was chosen. ISR was computed for each of the 30 segments as the number of IS divided by the duration of one segment (1/30th of the total NREM-cycle).

### Spectral Analysis and Instability Indices

Channels with IS, interictal bursts of low-amplitude fast activity (as identified by visual analysis), and/or discontinuous background were excluded from spectral analysis. For each remaining channel, we computed the power spectrum over non-overlapping 5-s temporal bins using Welch’s spectral estimation method between 0.5 and 180 Hz with a resolution of 0.5 Hz. Delta power was defined as the sum of the power contained between (and including) 0.5 and 4 Hz; RDP was defined as the ratio of delta power over total (0.5–180 Hz) power; sigma power was defined as the sum of power between 12 and 16 Hz. For each of the 30 segments composing the NREM-cycle, the delta and sigma power were averaged over all channels and over all 5-s temporal bins contained within the segment.

**Figure 1:**
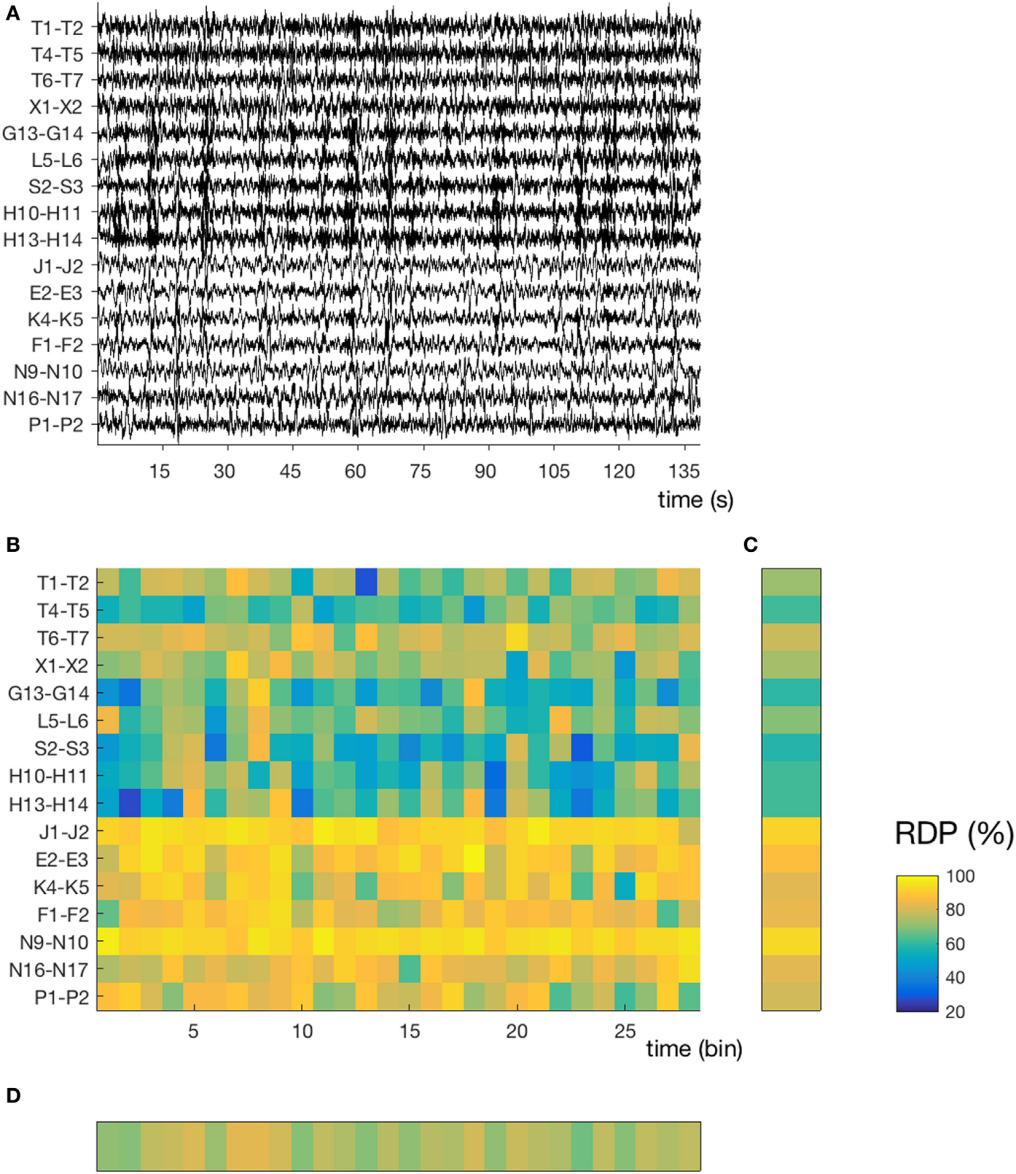
Temporal and spatial instability during unstable sleep (subject 4, segment 3). **(A)** Stereo-EEG traces in a bipolar montage without common contact; only contacts in the gray matter and without interictal spikes were considered for power analysis. In this segment (145 s during N2), we note a high spatial (nine channels with mixed activity, seven channels with predominantly delta) and temporal instability. The amplitude of each channel is normalized over time (z-score). **(B)** Relative delta power (RDP) computed on 5-s bins, represented as a matrix (channels × bins). **(C)** Temporal average of RDP (i.e., averaged over all bins for each channel independently), the SD of which defines the spatial instability **(D)** Spatial average (over all channels), the SD of which defines the temporal instability. For detailed location of contacts, see Supplementary Material.

**Figure 2:**
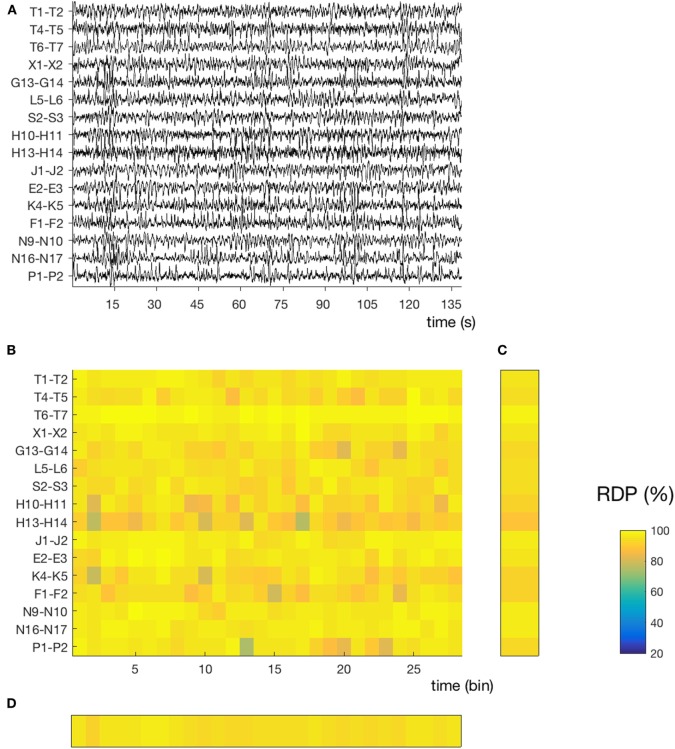
Temporal and spatial instability during stable sleep (subject 4, segment 27). **(A)** 145 s of sustained N3. Compared to the segment presented in Figure 1, we note that the EEG activity contains more slow waves and is more homogeneous over time and across channels. The same amplitude normalization as in the previous figure was used. **(B)** Relative delta power (RDP) computed on 5-s bins. **(C)** Temporal average of RDP (over all bins), the SD of which defines the spatial instability. **(D)** Spatial average (over all channels), the SD of which defines the temporal instability.

In addition to the (absolute) power in sigma and delta band, we defined two instability indices based on the spatial or temporal variation of RDP within each segment (Figures 1 and 2). The RDP spatial instability index was defined as the standard deviation (SD) over channels (“space”) of RDP averaged over all 5-s bins within a segment. Similarly, the RDP temporal instability was defined as the SD over all bins (“time”) after averaging the RDP over channels. We used the relative and not the absolute delta power in order to avoid influence of the different signal amplitudes between channels.

### Bivariate Correlation

The ISRs (both in the SOZ and in the DZ) in the 30 segments contained in each NREM-cycle were correlated with delta power, sigma power, RDP spatial instability, and RDP temporal instability using Spearman’s correlation. The significance level of the correlations was assessed by an exact permutation test. The statistical significance threshold was set to 0.05 after correction for false discovery due to multiple tests (i.e., correlation with each of the four EEG markers) with the Benjamini–Krieger–Yekutieli (BKY) procedure. All statistical tests were performed with built-in Matlab functions, except the BKY procedure, for which we used the code available in Ref. (30).

### Linear Model

A linear model was used in order to determine the relative importance of each EEG marker in describing the ISR. Since our EEG variables are not linearly independent (see Results), we used a so-called partial least square (PLS) model (31). PLS is a two-step linear model. During the first step, which resembles principal component analysis, the independent variables were combined into components in a way that both maximizes the variation between independent variables (EEG markers) and correlation with the depending variable (ISR in SOZ or DZ); during the second step, components were linearly combined to approximate the depending variable. The contribution of each independent variable was approximated as the sum of its relative contribution to each component multiplied by the variance explained by the component (31). For each dependent variable, the set of all its relative contributions (one for each patient) was compared with a distribution with zero-mean with a Wilcoxon rank sum test for equal medians (significance level was set to 0.05 after correction with the BKY procedure). Of note, delta and sigma power were log-transformed before use in the PLS model, in order to approximate a normal distribution (7). The PLS modeling was performed in Matlab (Statistics and machine learning toolbox).

## Results

### Patients

Ten patients (five females) fulfilled the inclusion criteria (one child, four adolescents, five adults); the mean age was 21.5 years (range 4–40). The first NREM-cycle was analyzed in five subjects, the second in two subjects, and the third, fourth, and fifth in one subject. The mean duration of the NREM-cycles was 54 min. See Table 1 for detailed demographic information.

**Table 1:** Patients’ demographics.

ID	Sex/age (years)	Epilepsy duration (years)	AED (mg/day)	Seizure-onset zone (SOZ)^a^	Diffusion zone (DZ)	Cycle Nb	Cycle duration (min)
1	m/4	2	TPM 100, CBZ 300 LTG 10	Operculum (precentral) R	Precentral gyrus R	1	72
2	f/14	9	OXC 2400, LEV 1000	F3 (pars triangul.)	F3	1	57.5
3	f/15	6	LEV 300, PB 125	F1–F2 L	F2 L	3	57
4	m/15	9	CBZ 600, PRM 750	Front. cingulate gyrus R	Operculum (central) R	2	67
5	f/16	9	OXC 1200, LEV 1000	F2–F3 L	Insula L	1	49.5
6	m/19	15	CBZ 900	F2 R	F1m R	2	40
7	f/27	11	CBZ 1200, PB 300	Precuneus R	T1 R	5	37
8	f/28	22	VPA 2000, OXC 1500	Insula R	Supramarginal gyrus R	1	44.5
9	m/37	17	CBZ 1600, TPM 300	F1m post R	F1m central R	1	72.5
10	m/40	33	VPA 500, TPM 500, OXC 1200, CNZ 2, LTG 300	F1m central R	Anterior cingulate gyrus R	4	45

*AED, seizure-suppressant drug; CBZ, carbamazepine; CNZ, clonazepam; F1–F2, superior frontal sulcus; F1m, mesial part of the superior frontal gyrus; F2, middle frontal gyrus; F2–F3, inferior frontal sulcus; F3, inferior frontal gyrus; L, left; LEV, levetiracteam; OXC, oxcabazepin; PB, phenobarbital; PRM, primidone; R, right; T1, superior temporal gyrus; TPM, topiramate; VPA, valproic acid*.

*^a^For all patients, the focal cortical dysplasia was located at the SOZ*.

### Dynamics of Individual EEG Variables

**Figure 3:**
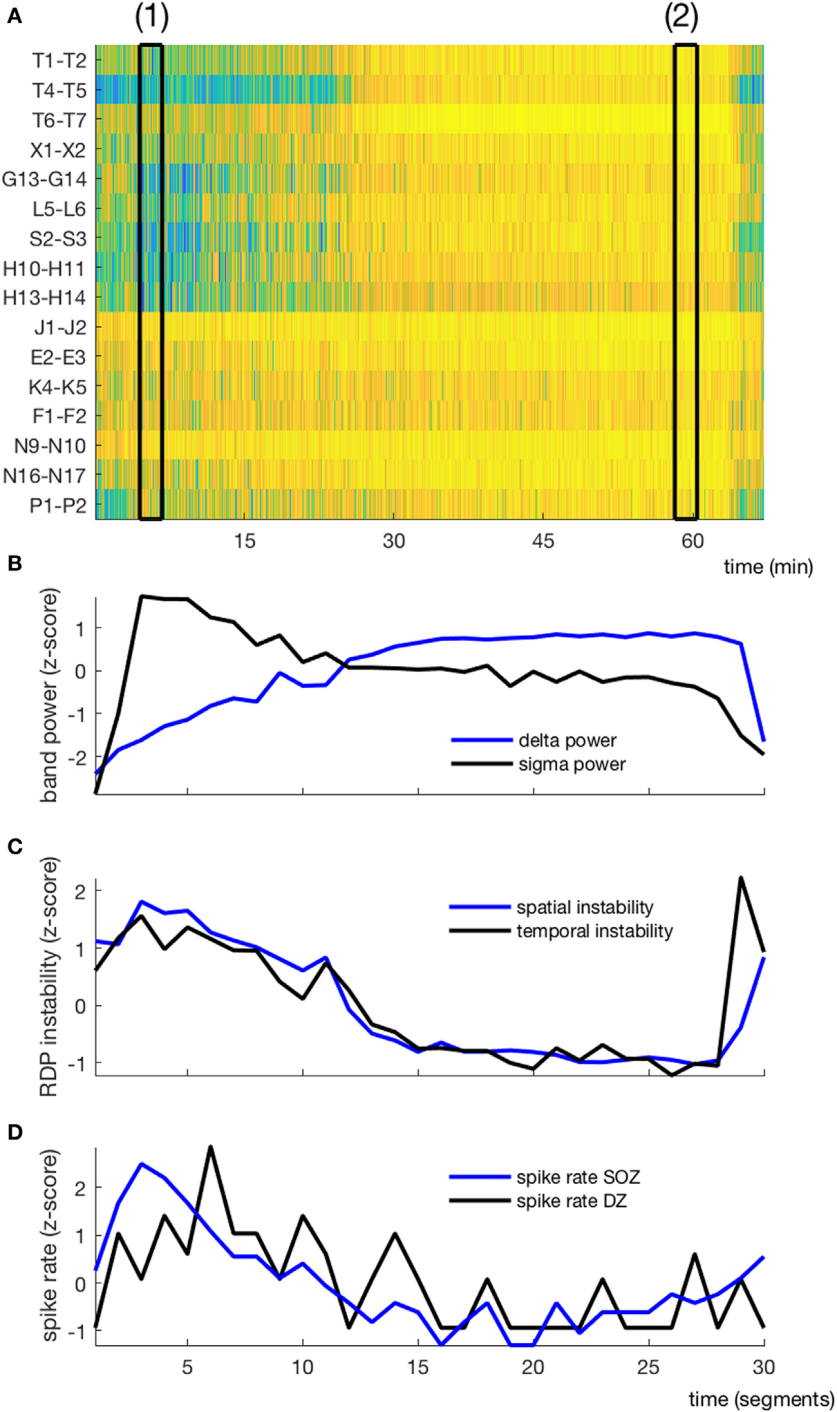
EEG markers and interictal spikes during one non-rapid-eye-movement-cycle in a typical patient (subject 4). **(A)** Relative delta power in spike-free channels represented as a matrix (channel × time); black rectangles (1) and (2) correspond to the segments presented in Figures 1 and 2, respectively. **(B)** Temporal course of absolute delta (blue) and sigma (black) power; the cycle was decomposed into 30 segments, over which the sigma and delta power were averaged. **(C)** Temporal course of spatial (blue) and temporal (black) instability. **(D)** Temporal course of interictal spike rate in the seizure-onset zone (blue) and the diffusion zone (black). No averaging window between data points.

**Figure 4:**
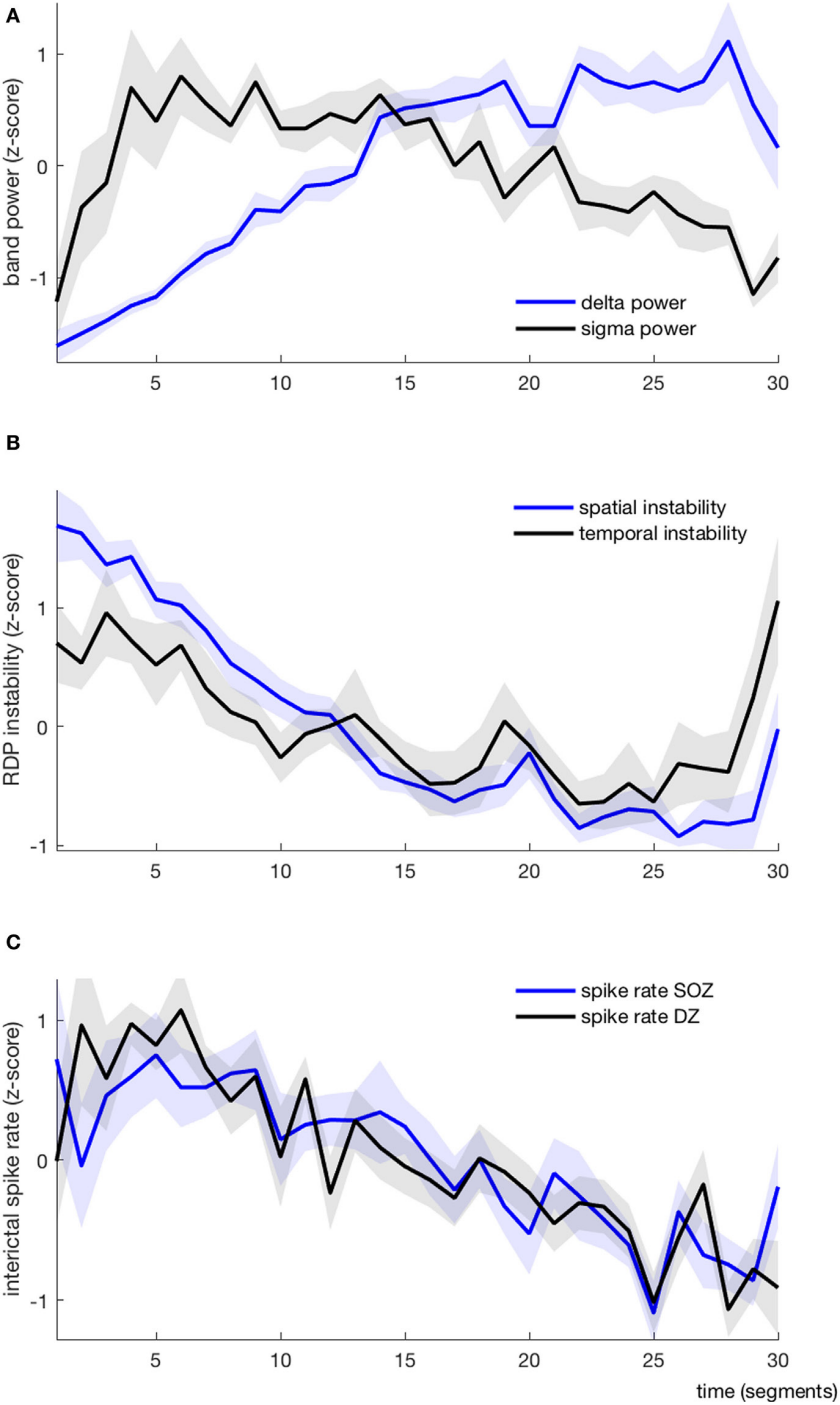
EEG markers and interictal spikes during one non-rapid-eye-movement-cycle, average over all patients (each variable is represented as mean ± SEM). **(A)** Absolute delta (blue) and sigma (black) power. **(B)** Spatial (blue) and temporal (black) instability **(C)** Interictal spike rate in the seizure-onset zone (blue) and the diffusion zone (black).

Each NREM-cycle was decomposed into 30 non-overlapping segments, over which we averaged the four EEG parameters and computed the ISR in the SOZ and DZ. The temporal course of the different variables during one NREM-cycle for a representative patient (Subject 9) is presented in Figure 3. The averaged dynamics (mean of 10 patients ± SEM) is shown in Figure 4.

#### Absolute Band Power

As expected, the delta power was systematically lower at the beginning of the NREM-cycle (we considered for each subject the index (from 1 to 30) of the segment with the minimal value of delta power; the median value for this index was 1.5, the range was 1–3) (Figure 4A). Delta power generally increased progressively over time and reached its maximum value shortly before the end of the cycle (median segment index = 26.5, range = 19–29), after which it usually dropped again in the very last segment(s). Usually, the sigma power was also relatively low at the beginning of the cycle; it then increased (more rapidly than the delta power) and reached its maximum usually within the first quarter of the cycle (median segment with maximal sigma power = 4, range = 1–15), after which it started to slowly decay. In 7/10 patients, the sigma power increased again in the very last segment(s). As such, the delta and sigma power were usually moving along the same direction in the very first segments and in a few patients also in the last segments, whereas in-between, during approximately 2/3 of the cycle, the two curves had opposite directions.

#### Instability Indices

**Figure 5:**
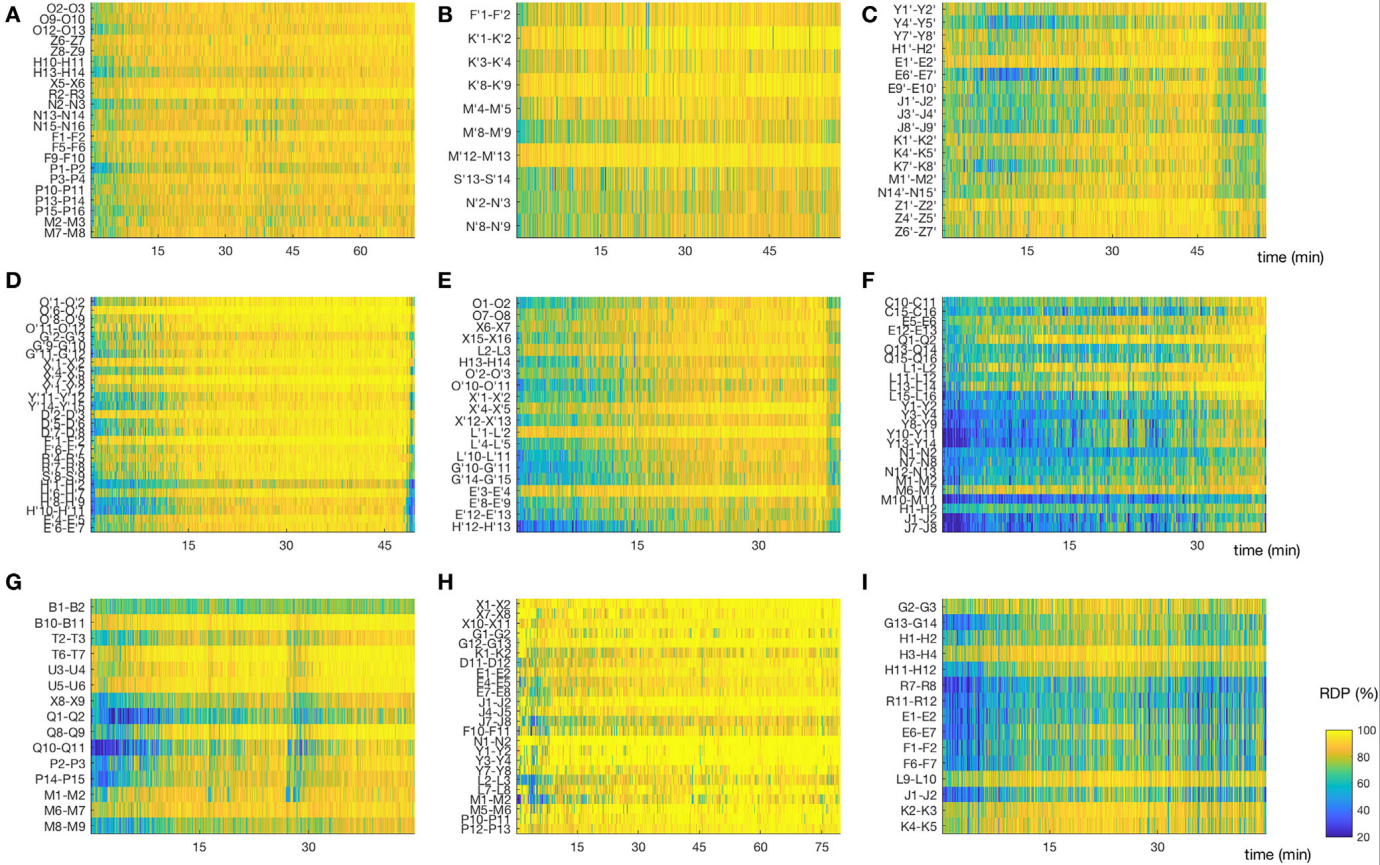
Matrix of relative delta power (RDP). The non-REM-cycle of subjects 1–3, 5–10 (for subject 4, see Figure 3A) represented as a matrix (channel × time) of RDP. The same color scale is used for all patients. **(A,B,D)** Subjects 1, 2, and 5, in whom the first non-rapid-eye-movement (NREM)-cycle was analyzed; we observed a regular increase of RDP during most of the cycle. **(C,F,I)** Subjects 3,7, and 10. For these patients, we did not analyze the first NREM-cycle. As expected, the overall level of RDP is reduced, although some channels do reach a high percentage of delta activity. **(E)** Subject 6, for whom the second cycle was analyzed; we observed nonetheless a relative global progression of RDP. **(G)** Subject 9, first cycle. This patient had a non-monotone build-up of delta activity. Interestingly, the spike rate in both the seizure-onset zone and the diffusion zone increased during the local troughs of RDP. **(H)** Subject 9, first cycle. The EEG was recorded after sleep deprivation, and the patient went very rapidly into stage N3; note the almost immediate appearance of homogeneous and stable high RDP (For detailed location of contacts, see Supplementary Material).

We derived two instability indices based on the value of RDP over time and channels during the NREM-cycle (Figures 3A and 5).Both instability indices were relatively high at the beginning of the cycle and decreased during the major part of the cycle, reaching their minimum shortly before the end of the cycle (median segment for minimal spatial instability = 26, range = 16–29; median for temporal stability = 24.5, range = 10–28), after which the indices increased rapidly (Figure 4B). For all patients, the spatial instability was the highest at the beginning of the cycle (median segment: 1), whereas the temporal instability was more variable, usually highest at the end (maximum value within the last four segments in half of the patients; median segment with maximum = 22.5, range = 1–30). Both instability indices were negatively correlated with delta power in all patients. However, the degree of correlation was different among patients, so RDP instability indices brought additional information with respect to delta power (see for instance subject 3, Table 2).

**Table 2:** Bivariate Spearman correlation between EEG markers and interictal spikes in the seizure-onset zone (*p < 0.05; **p < 0.01 after correction for multiple tests).

Subject	Power		Relative delta power (RDP) instability	
	Delta	Sigma	Spatial	Temporal
1	−0.50**	0.46**	0.76**	0.71**
2	0.62**	0.54**	−0.54**	−0.01
3	−0.19	0.61**	0.65**	0.42*
4	−0.75**	0.33*	0.76**	0.77**
5	−0.84**	0.67**	0.73**	0.73**
6	−0.31	0.05	0.30	0.14
7	−0.80**	0.24	0.75**	0.15
8	−0.32	0.33	0.35	0.25
9	−0.41**	0.81**	0.84**	0.80**
10	0.01	0.38*	−0.18	−0.03

#### Interictal Spike Rate

The ISR was typically at the highest shortly after the beginning of the cycle (median segment with maximal SR in the SOZ = 4.5, range = 1–22; median segment with maximal SR in the DZ = 5.5, range = 1–18). The total number of spikes was higher during the first than during the second half of the NREM-cycle in 9/10 patients when counted in the SOZ and in 10/10 patients for the DZ.

### Spearman Correlation

In the SOZ (Table 2), the rank correlation (Spearman’s rho) between delta power and ISR was negative for 8/10 subjects (in 5/8 cases, the negative correlation reached statistical significance after correction for multiple tests). The correlation between IS and delta power was positive in the remaining two subjects (one adolescent and one adult, for the former reaching statistical significance); these two patients were not presenting different demographic or electrophysiological parameters. The correlation between sigma power and ISR was positive in all patients, reaching statistical significance in seven cases. The spatial RDP instability had a positive rank correlation with IS in 8/10 cases (6 were significant) and a negative correlation in 2/10 (1/2 were significant). The temporal RDP instability was positively correlated in 8/10 cases (5/8 significant) and negatively correlated in the remaining 2 (0/2 significant).

Results were qualitatively similar for the spike rate measured in the DZ (Table 3). The rank correlation with delta power was negative in all patients (7/10 significant), the correlation with sigma power was positive in 9/10 patients (5/8 significant), and the correlation with spatial and temporal RDP instability was positive in all subject (significant in 7 and 6 subjects, respectively).

**Table 3:** Bivariate Spearman correlation between EEG markers and interictal spikes in the diffusion zone (*p < 0.05, **p < 0.01 after correction for multiple tests).

Subject	Power		Relative delta power (RDP) instability	
	Delta	Sigma	Spatial	Temporal
1	−0.63**	0.69**	0.88**	0.61**
2	−0.40*	0.17	0.58**	0.39*
3	−0.34*	0.59**	0.74**	0.53**
4	−0.50**	0.56**	0.53**	0.59**
5	−0.22	0.34	0.19	0.10
6	−0.19	−0.003	0.23	0.05
7	−0.67**	0.28	0.65**	0.29
8	−0.51**	0.23	0.58**	0.60**
9	−0.44**	0.54**	0.68**	0.60**
10	−0.14	0.38*	0.03	0.27

### Linear Model

**Figure 6:**
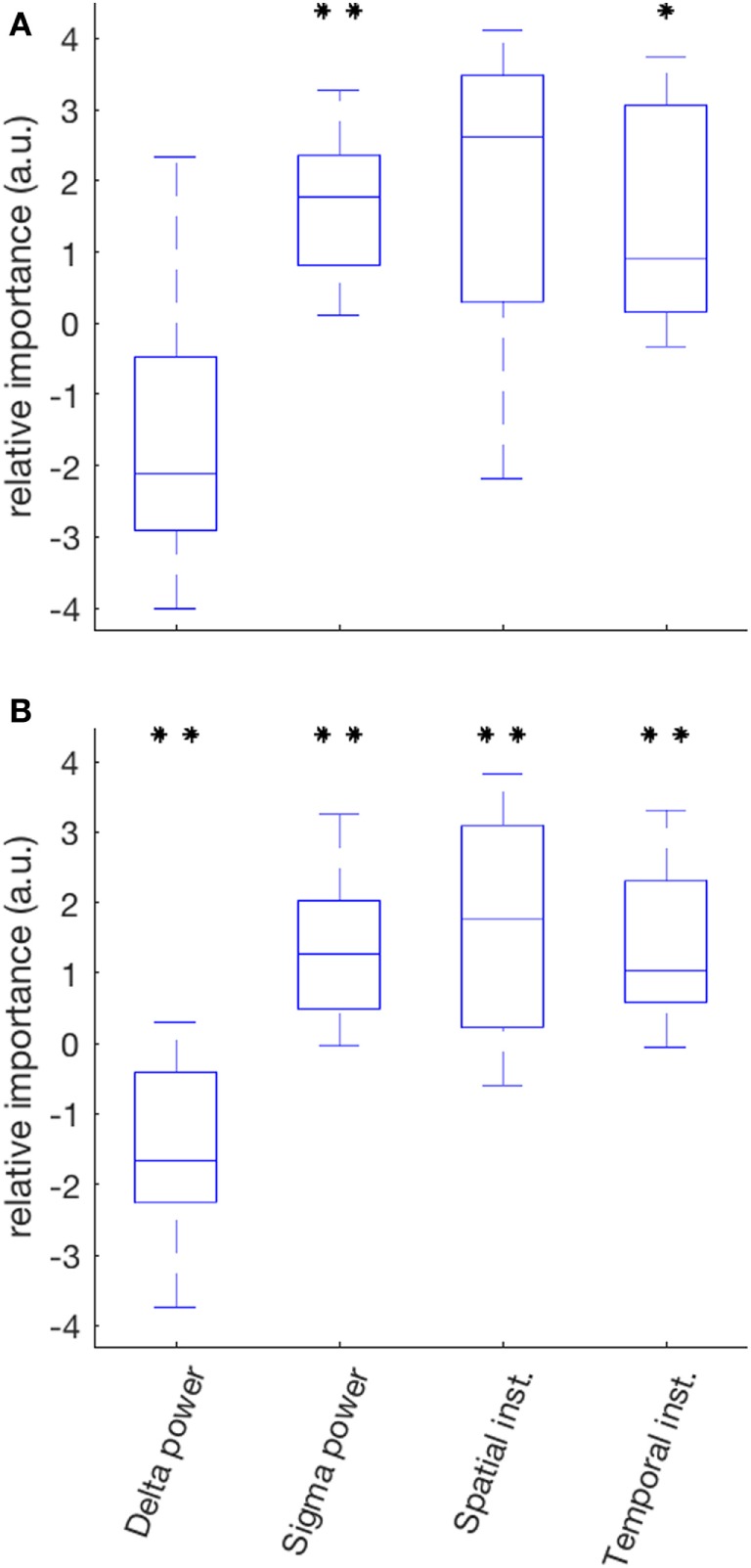
Relative importance of the four EEG markers in predicting the IS rate in a two-step linear model (partial least square). **(A)** In the seizure-onset zone, the relative contribution of sigma power over all patients was the most significantly different from a distribution with zero-mean (positive correlation); the other markers with a statistically significant contribution was temporal instability. **(B)** In the diffusion zone, all predictors had a contribution significantly different from zero: negative correlation for delta power, positive for sigma power, spatial instability and temporal instability (*p < 0.05; **p < 0.01 after correction for multiple test).

The median variance of ISR in the SOZ explained by the linear model was 55% (range 14–91%); sigma power was the only EEG variable with a positive contribution in all 10 patients (Figure 6A). It was also the variable for which the set of relative contributions (one value for each patient) was the most significantly different from a distribution with zero-mean (*p* = 0.008). The other variable with a set of relative contributions statistically different from a zero-mean distribution was the temporal instability of RDP (*p* = 0.04).

The median variance of ISR in the DZ explained by the linear model was 44% (range 11–76%); for each EEG variable, the set of its contribution was significantly different than a distribution with zero-mean; the relative contributions were mainly negative for delta power (*p* = 0.008) and positive for sigma power (*p* = 0.008), spatial instability (*p* = 0.098), and temporal instability (*p* = 0.008) (Figure 6B).

## Discussion

In this work, we compared the ISR both within and outside the SOZ with four EEG characteristics during a single NREM-cycle in 10 patients with focal epilepsy due to a type 2 FCD.

We found that the ISR was positively correlated with the power in the sigma frequency band. Interestingly, Clemens et al. (12) found a higher ISR during N2 than during N3 in foramen ovale recordings in patients with mesio-temporal epilepsy, the stage where spindle activity is higher. It also corroborates previous studies showing that ISR and sigma power are correlated in various childhood epilepsy syndromes (7, 8, 21). A relationship between sleep spindles and IS has been previously recognized in clinical and experimental works. It has been postulated, for instance, that certain types of generalized spike-waves are expression of an abnormal response to the volley of thalamocortical excitatory inputs usually producing sleep spindles (17, 18, 32–34). It is unclear if this mechanism applies to FCD2, since the mechanism linking spindles and spike production could be different in focal than in generalized epilepsy. For instance, the causal relationship could also be in the opposite direction: Gelinas et al. showed that that IS are correlated with and induce cortical spindle-like responses both in an animal model and in patients with focal epilepsy undergoing pre-surgical evaluation with electrocorticography (35).

By contrast, delta power was negatively correlated with ISR in the majority of subjects. Our results are at odds with previous works describing a positive correlation between ISR and delta power in adult patients with focal epilepsy (6, 21). However, these studies were conducted on scalp EEG, whereas we used intracranial EEG. This difference is important, as works using simultaneously extra- and intracranial recording have shown that only a fraction of IS are detected on the scalp (12, 13). The reason why IS seem to propagate better during deep sleep remains unclear. Even though NREM sleep is known to increase the (local) amplitude of the cortical response to a stimulus (36), which could be a candidate explanation, we did not find a consistent association between spike amplitude and delta power in our patients (data not shown). Of note, none of the 10 patients did present clear spikes or spike-waves in the extracranial EEG channels (see Supplementary Material).

Frauscher et al. (23) showed that ISR in intracerebral recordings was higher during high amplitude and widespread slow waves than in segments serving as control, chosen within the same sleep stages. However, the authors analyzed single waves and did not treat separately waves during sustained high level of delta activity from waves occurring during oscillatory patterns (see below).

We evaluated the influence of delta activity on the ISR not only as a function of its absolute power but also in relation with the spatial and temporal variation of its relative power. To quantify the latter, we introduced a new measure called “temporal instability,” which was defined as the standard variation of RDP over a given time segment. This measure is influenced both by monotone variations (e.g., during the gradual “descent” to deep sleep) and by transient changes in the relative importance of power of delta, such as measured with cyclic alternating patterns or other types of oscillations (22, 37). We found that the RDP temporal instability was positively correlated with ISR. These results are in line with studies showing that both a high increase rate of delta power (6) and alternating periods of high and low delta activity (22, 38–40) favor the production of epileptic activity. Similarly, we defined the “spatial instability” as the standard variation of RDP across the different channels. The motivation for using this measure was the well-known fact that delta-sleep can be a local process (13–15). Here also, we found a positive correlation between instability and ISR. To the best of our knowledge, this is the first time that spatial variation of delta activity in sleep is quantified in the context of epileptic activity.

Our data show that the lowest ISR during the NREM-cycle was found during the plateau of delta activity, corresponding to a stable and homogeneous production of delta activity in different brain regions. We know from single unit recording studies conducted in humans that during this part of the sleep cycle, individual neurons are maximally synchronized [or more exactly, their firing pattern is maximally coordinated by alternating global up and down states (14)]. One can postulate that during this very regulated phase, neurons are less likely to enter a peculiar firing pattern as observed during IS (2). In this context, the fact that ISR occurred predominantly during the transition from up to down states in the study of Frauscher et al. could be in part due to a permissive effect, rather than to a facilitating effect (that is, global up and down states suppress IS, and the transition between the states becomes the only moment where an IS is “allowed” to occur).

This interpretation is consistent with the experimental and theoretical works suggesting that large-scale synchronization might act as a suppressor for epileptic activity (41), as it prohibits a single region to develop an autonomous, non-physiological activity. Interestingly, the negative correlation with delta power and positive correlation with RDP instability was found in more patients in the DZ than in the SOZ. Of course, this observation was made only on a small number of subjects; however, it is in line with an older study in which regions close to the SOZ were found to be less influenced by the transition between wakefulness to NREM and NREM to REM (the power in different frequency bands was not analyzed) (5). If confirmed, this difference could be related to the fact that the SOZ has been shown to be “functionally disconnected” from the rest of the brain network (42–44). It would thus be less sensitive to large-scale events (such as slow waves) than the DZ, which is constituted of more “healthy” tissue. Similarly, the relative influence of the thalamocortical oscillations producing spindles could be higher in the SOZ due to the fact that it acts locally on hyper-excitable dysplastic neurons. Finally, it is worth mentioning that due to its peculiar cyto-architecture and electrophysiological properties, the functioning of FCD2 during sleep has been compared to the behavior of the reticular-thalamic nucleus, the “pace-maker of sleep spindles” (45). It seems plausible that the mechanisms responsible for the production of spindle activity in the reticular nucleus act simultaneously on the core of the FCD, stimulating the production of IS.

### Instability Measures

The representation of sleep in a matrix of RDP (Figure 5) and the two instability indices we introduced could prove useful to study sleep physiology even in the absence of epilepsy. They offer a compact description of the local production of slow wave activity, the marker of sleep homeostasis. We noted, for instance, that at the beginning of N2 (as detected on the scalp EEG), some channels were already containing a high proportion of delta power. The gradual increase of absolute delta power (measured at a global level) was accompanied by a homogenization of RDP at the local level, leading to a reduction of spatial instability. This non-homogeneous aspect of sleep onset, whereby some brain regions present sleep-like EEG activity before others, was already described in the neocortex with respect to the hippocampus (46) or the thalamus (47). More recently, Slater et al. confirmed these observations using a quantifying method based on the number of channels that were considered at sleep in electrocortigographic data (48). Our approach has the advantages of being easier to compute and of allowing further characterization of EEG recording during sleep. Of note, both instability indices were based on a bipolar montage without common contact; we did not investigate how these indices would have been affected by the choice of another montage.

### Strengths and Limitations

We are confident in the validity of our results, since we performed our analysis on intracerebral EEG and ISR. The fact that none of the subjects had clear IS in the co-registered extracranial EEG channels and only one subject had possible IS in the previously performed 10:20-EEG confirms the superiority of intracerebral EEG, at least in the present group of patients. In addition, we considered not sleep stages [which are coarse and rigid segmentation of sleep (22)] but markers derived from spectral analysis, that is, with continuous variables better reflecting the dynamics of sleep.

One possible limitation of our study is the fact that we incorporated only patients with FCD2. It is not clear how the findings would generalize to other focal epileptic syndromes. However, the similarity of our results with those of Clemens et al. (12) conducted with intracranial electrodes in patients with mesial temporal lobe epilepsy (rarely associated with FCD2) is a possible indication that our observations are independent of the histopathological substrate.

Another potential limitation is the fact that we used the first NREM-cycle in only half of the patients. However, we did not find a statistical difference in the correlation values between absolute band power and ISR between these patients and the ones where a subsequent cycle was used (SOZ: *p* = 0.98 for difference in correlation of IS with sigma power, *p* = 0.34 for delta power; DZ: *p* = 1 for sigma and delta power; Wilcoxon rank sum test), even though the overall RDP value was reduced in the latter (Figure 5).

The homeostatic pressure was not taken into account in our multimodal model. Future works could compare the ISR before and after sleep deprivation or compare several different sleep cycles within patients.

### Conclusion and Outlook

This study contributes to clarify the influence of slow waves, sleep spindles, and sleep instability on ISR during NREM sleep. Our findings could be of clinical relevance, for instance, for enforcing and monitoring the effects of therapy in patients suffering from epilepsy and sleep disorder, to evaluate the effects or side effects of pharmacological treatment, or for future protocols of non-invasive stimulations.

The focus of this study was the relation between EEG markers and ISR during NREM sleep, and therefore, we did not investigate the transition from wakefulness to sleep. Future work will include applying this method to sleep onset, possibly comparing the dynamics of instability computed on high-resolution scalp EEG and on intracranial EEG.

## Ethics Statement

This study was carried out in accordance with the recommendations of the Ethics Commission of the Ospedale Niguarda. All patients gave written informed consent for retrospective analysis of their data in accordance with the Declaration of Helsinki.

## Author Contributions

FZ, KS, and LN designed the study; AR, GR, and LN acquired the data; FZ and LN performed the analysis; all authors wrote the manuscript.

## Conflict of Interest Statement

The authors declare that the research was conducted in the absence of any commercial or financial relationships that could be construed as a potential conflict of interest.
