# Supplementary material for: Correlating Interictal Spikes with Sigma and Delta Dynamics during Non-Rapid-Eye-Movement-Sleep
Source: Front Neurol. 2017 Jun 22;8:288. doi: 10.3389/fneur.2017.00288 (PMC5479894; doi:10.3389/fneur.2017.00288)
Supplement: Supplementary file 1 [file presentation_1.pdf]

Supplementary Material: Correlating Interictal Spikes with Sigma and Delta Dynamics during NREM-Sleep

Frederic Zubler, Annalisa Rubino, Giorgio Lo Russo, Kaspar A. Schindler, Lino Nobili*

*Correspondence:
Author Name: Lino Nobili
lino.nobili@ospedaleniguarda.it

1 MONTAGE

All montages used for the analysis of extracellular field recording have advantages and disadvantages. One of the main characteristics of the bipolar montage is that it cancels out the common activity (= simultaneous and of same amplitude) present at neighboring contacts. This effect can be detrimental when the voltages recorded result from locally produced neural activity. On the other hand, cancelation of perfectly synchronous activity at neighboring contacts is beneficial in that it removes field effects, as well as the possible activity of the reference electrode, which is rarely truly inactive. This important point is illustrated in Figure S1, demonstrating than in a bipolar montage the contrast between different brain regions can be increased.

2 EXTRACRANIAL VS. STEREO-EEG

2.1 Epileptiform activity in extracranial EEG

The interictal epileptic activity is usually very discrete in patients with FCD2. From our 10 patients, only one had interictal epileptiform activity in the 10:20 standard extracranial EEG prior planning of the Stereo-EEG (subject 1, "possible spike-waves frontal right"). In the extracranial channels recorded simultaneously with SEEG (usually Fz and Cz), we did not observe typical spike-waves in this patient, neither in any other patient. Episodically, some sharply contoured transient occurred simultaneously to IS observed in the intracerebral recording (see for instance Figure S2). However, these transients could not be recognized reliably by 2 different electroencephalographers blinded to the intracerebral traces.

2.2 Power analysis in extracranial EEG

We computed the delta and sigma power, as well as the relative delta power (RDP) temporal instability on a single channel in the extracranial EEG (Fz, chosen because this electrode was available for all patients) using the same procedure than for SEEG (see main text). The spatial instability could not be computed on the scalp because we did not have enough channels during the co-registration.

The time course of the EEG markers (delta, sigma, temporal instability) averaged over the 30 segments of the NREM-cycle was qualitatively similar to the results obtained using SEEG. Figure S3 shows the results for a single patient (subject 4; compare with Figure 3 in the main text); Figure S4 shows the average over all patients (compare with Figure 4 in the main text). Note however that the temporal instability had

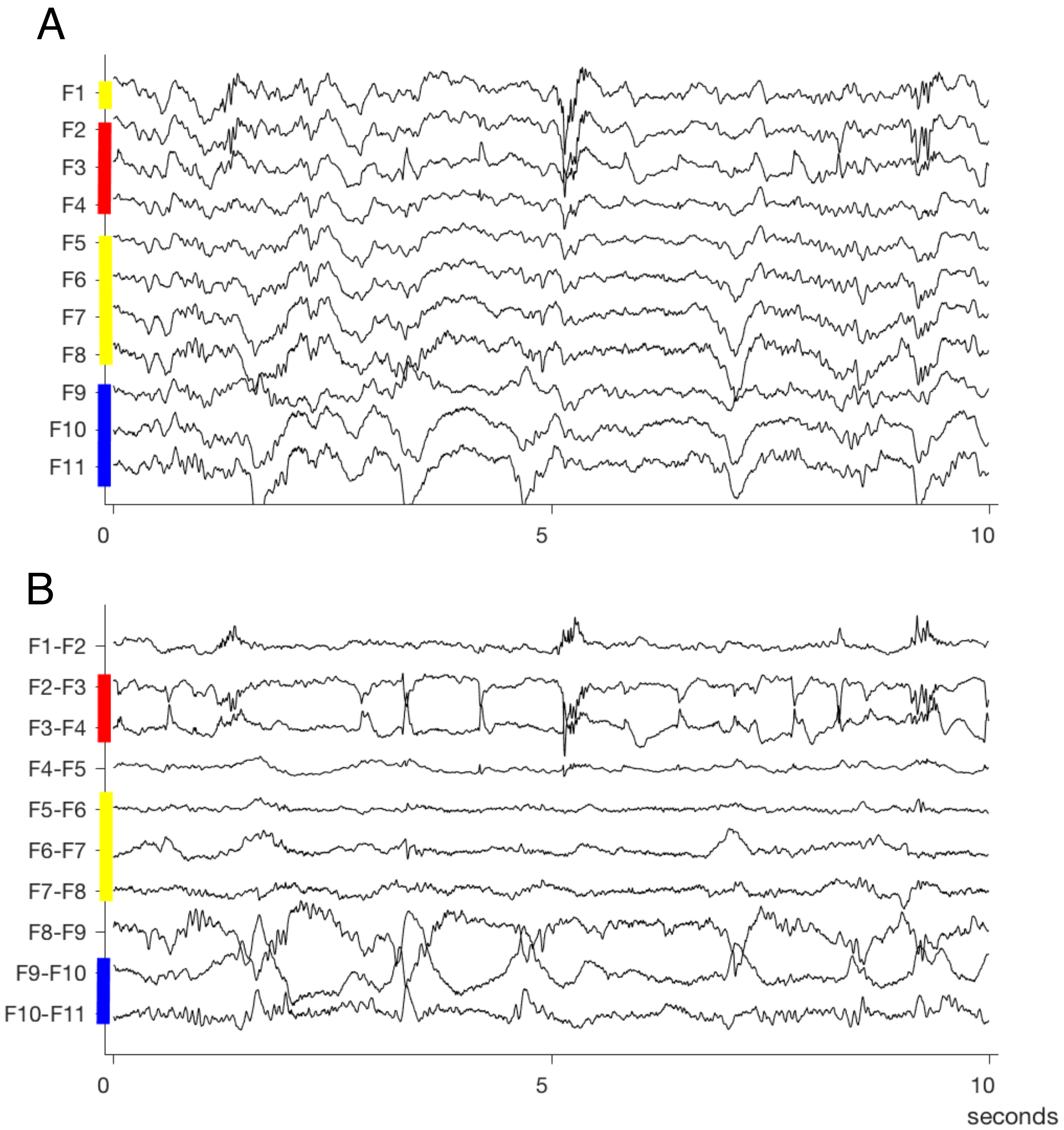

Figure S1. Differences between monopolar (A) and bipolar (B) montage. The figure represents a 10-second epoch registered by a single electrode (subject 9). Contacts near the focal cortical dysplasia are marked in red, contacts in the white matter in yellow, contacts in the neo-cortical grey matter in blue. By eliminating the common activity between neighboring channels the bipolar montage increases the contrast between zones with different histological substrates. In particular, the background activity in the white matter and near the lesion is reduced.

a wider spread. In general all power analysis performed on the scalp showed more noise, i.e. a higher temporal fluctuation (results not shown).

The Spearman correlation between the delta power at Fz and the ISR in the SOZ was very similar to the results obtained with the intracranial power (Table S1). The correlation was negative for 8/10 patients, and positive for two patients (subjects 2 and 13; who also showed a positive correlation when power was computed in SEEG). Moreover, the comparison of ranks of patients between extracranial and intracranial EEG (i.e. do patients with high correlation coefficient in one condition also show a high correlation in the other condition?) was high (Kendall's tau = 0.78). The Spearman correlation between delta power and ISR

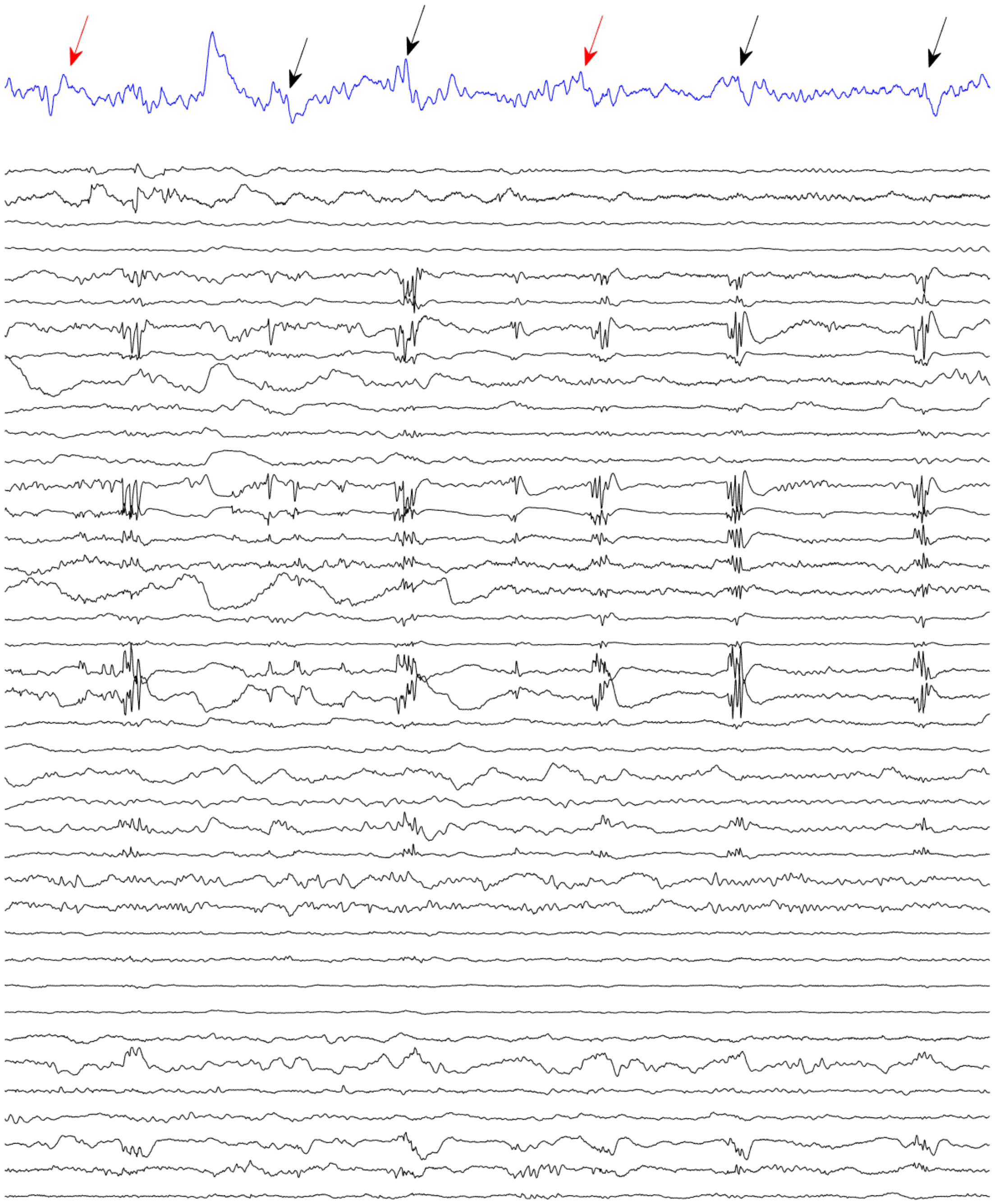

Figure S2. Co-registratation of extracranial EEG (channel Fz; blue) and Stereo-EEG (bipolar montage; black). The figure shows a 10-second epoch registered in subject 7; some sharply configured transients registered on the scalp were associated with IS in the SEEG (black arrows), whereas other transients, although very similar, occurred in absence of IS (red arrows).

in the DZ was also qualitatively very similar, all patients having a negative Spearman correlation, as was the case with intracranial power (Table S2).

By contrast, the correlation between sigma power in Fz and ISR in Stereo-EEG did change polarity for two subjects (in one of which the negative correlation was statistically significant); accordingly, the correspondance of ranks in correlation strength between results in SEEG and in extracranial EEG is lower

Table S1. Bivariate Spearman correlation between extracranial EEG markers and interictal spikes in the SOZ (*: p<0.05; **: p< 0.01 after correction for multiple test).

Subject	Delta power	Sigma power	Temporal instability
1	-0.17	0.22	0.72**
2	0.5**	0.34	0.07
3	-0.24	0.59**	0.56**
4	-0.71**	0.47**	0.74**
5	-0.81**	0.18	0.41*
6	-0.21	0.07	0.30
7	-0.70**	-0.68**	-0.11
8	-0.3	0.28	0.007
9	-0.56**	0.38*	0.48**
10	0.1	0.44**	0.15

(Kendall's tau = 0.24) than for delta power. The most likely explanations is the noise in sigma frequencies in the extracranial recording. We cannot exclude an additional weaker propagation of sleep spindles, but did not systematically explore this effect.

The correlation between RDP temporal instability in extracranial EEG and ISR in Stereo-EEG had the same polarity than when computed in intracranial EEG in 8/10 patients (Kendall's tau = 0.47).

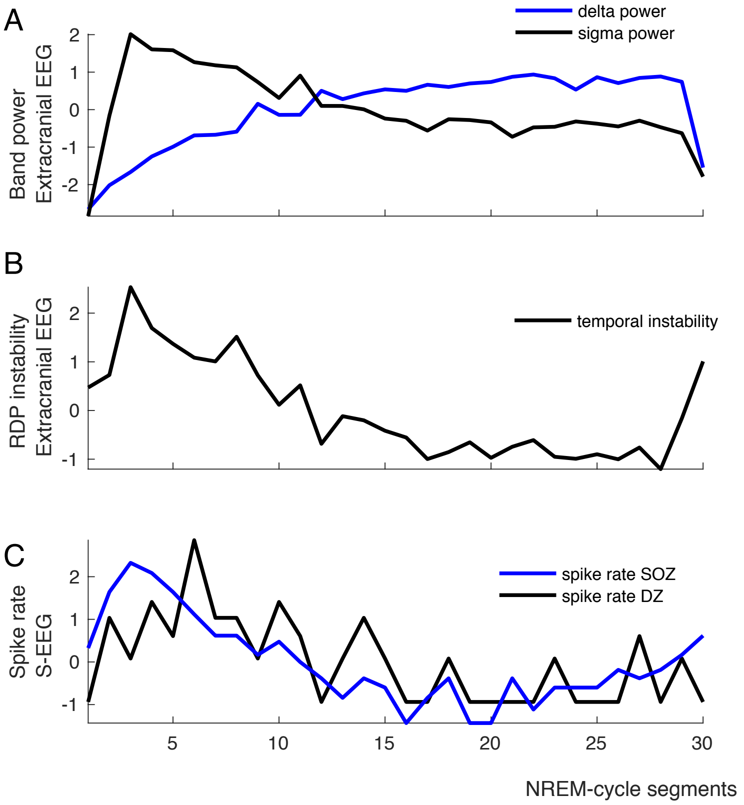

Figure S3. EEG markers form the extracranial recordings and ISR from the SEGG for a single patient (subject 7). (A) Absolute delta and sigma power computed on Fz. (B) Temporal instability computed from RDP on Fz. (C) ISR counted on SEEG.

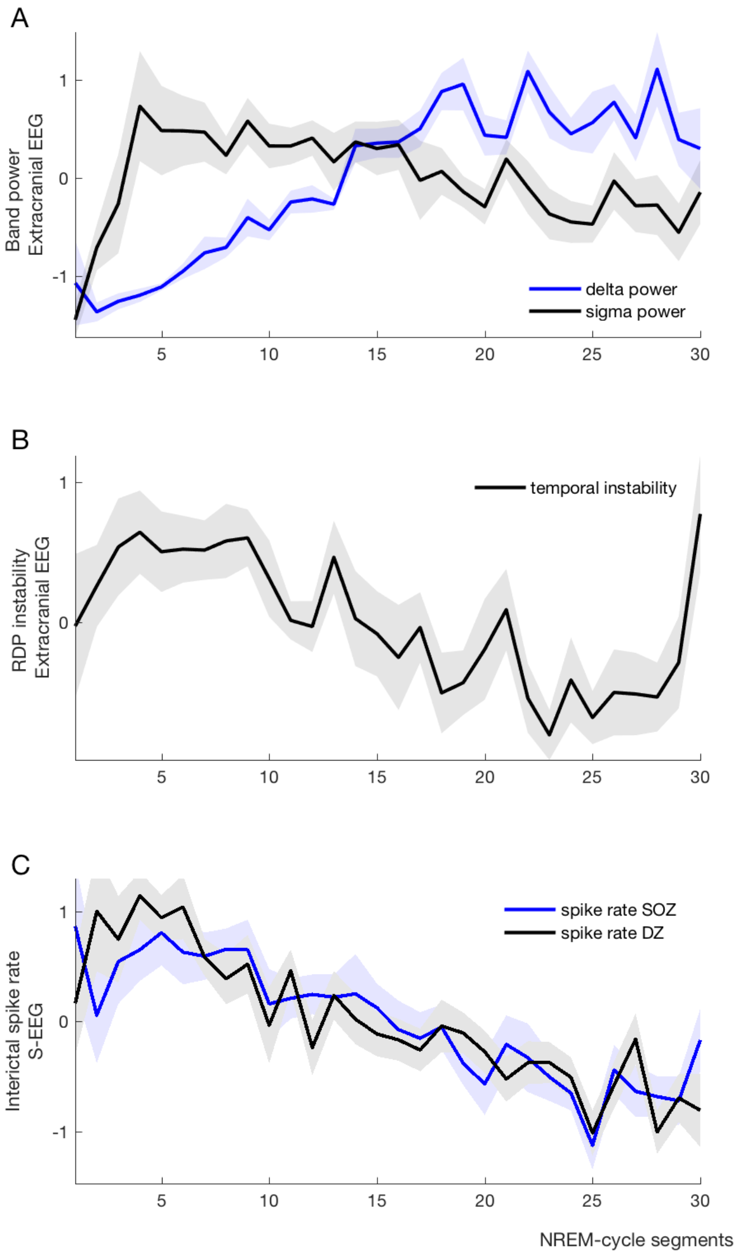

Figure S4. EEG markers form the extracranial recordings and ISR from the SEGG averaged over all patients. (A) Absolute delta and sigma power computed on Fz. (B) Temporal instability computed from RDP on Fz. (C) ISR counted on SEEG

Table S2. Bivariate Spearman correlation between extracranial EEG markers and S-EEG interictal spikes in the DZ (*: $p<0.05$; **: $p< 0.01$ after correction for multiple test).

Subject	Delta power	Sigma power	Temporal instability
1	-0.31	0.46*	0.65**
2	-0.51**	0.02	0.38
3	-0.41*	0.53**	0.73**
4	-0.50**	0.73**	0.64**
5	-0.27	-0.00	0.08
6	-0.20	-0.10	0.14
7	-0.54**	-0.70**	-0.05
8	-0.40*	0.44*	0.32
9	-0.54**	0.27	0.46*
10	-0.01	0.21	0.34

Electrode position

Each SEEG-electrode is identified with a letter, each contact on the electrode with a number. The numbering is ascending from internal to external (1 being the deepest contact). The list below gives the detailed location of each pair of contacts.

Subject 1

O2-O3	Central cingulate gyrus R
O9-O10	Inferior frontal gyrus R
O12-O13	Inferior frontal gyrus R
Z6-Z7	Inferior frontal gyrus R
Z8-Z9	Inferior frontal gyrus R
H10-H11	Middle frontal gyrus R
H13-H14	Middle frontal gyrus R
X5-X6	Inferior frontal gyrus R
R2-R3	Insula (short gyri) R
N2-N3	Frontal cingulate gyrus R
N13-N14	Precentral gyrus (inferior part) R
N15-N16	Precentral gyrus (inferior part) R
F1-F2	Frontal cingulate gyrus R
F5-F6	Superior frontal sulcus R
F9-F10	Superior frontal gyrus (lateral part) R
P1-P2	Posterior cingulate gyrus R
P3-P4	Posterior cingulate gyrus R
P10-P11	Postcentral gyrus R
P13-P14	Postcentral gyrus R
P15-P16	Postcentral gyrus R
M2-M3	Superior frontal gyrus (mesial part) R
M7-M8	Superior frontal gyrus (mesial part) R

Subject 2

F'1-F'2	Superior frontal gyrus (lateral part) L
K'1-K'2	Frontal cingular gyrus L
K'3-K'4	Frontal cingular gyrus L
K'8-K'9	Superior frontal gyrus (lateral part) L
M'4-M'5	Superior frontal gyrus (lateral part) L
M'8-M'9	Middle frontal gyrus posterior L
M'12-M'13	Middle frontal gyrus posterior L
S'13-S'14	Pre-central gyrus L
N’2-N’3	Paracenetral lobule L
N'8-N'9	Paracenetral lobule L

Subject 3

Y1'-Y2'	Superior frontal gyrus (mesial part) L

Y4'-Y5'	Superior frontal gyrus (lateral part) L
Y7'-Y8'	Superior frontal gyrus (lateral part) L
H1'-H2'	Superior frontal sulcus L
E1'-E2'	Superior frontal gyrus (mesial part) L
E6'-E7'	Medial frontal gyrus L
E9'-E10'	Medial frontal gyrus L
J1'-J2'	Superior frontal gyrus (mesial part) L
J3'-J4'	Superior frontal gyrus (mesial part) L
J8'-J9'	Superior frontal gyrus (lateral part) L
K1'-K2'	Superior frontal gyrus (mesial part) L
K4'-K5'	Superior frontal gyrus (lateral part) L
K7'-K8'	Superior frontal gyrus (lateral part) L
M1'-M2'	Medial part of superior frontal gyrus L
N14'-N15'	Precentral gyrus L
Z1'-Z2'	Medial part of superior frontal gyrus L
Z4'-Z5'	Superior frontal gyrus (lateral part) L
Z6'-Z7'	Superior frontal gyrus (lateral part) L

Subject 4

T1-T2	Anterior insula R
T4-T5	Anterior insula R
T6-T7	Anterior insula R
X1-X2	Orbitofrontal cortex R
G13-G14	Inferior frontal gyrus R
L5-L6	Superior temporal gyrus R
S2-S3	Insula (short gyri) R
H10-H11	Inferior frontal gyrus (pars opercularis) R
H13-H14	Inferior frontal gyrus (pars opercularis) R
J1-J2	Superior frontal gyrus (lateral part) R
E2-E3	Superior frontal gyrus (lateral part) R
K4-K5	Superior frontal gyrus (lateral part) R
F1-F2	Superior frontal gyrus (lateral part) R
N9-N10	Inferior frontal gyrus (pars opercularis) R
N16-N17	Inferior frontal gyrus (pars opercularis) R
P1-P2	Parietal cingulate gyrus R

Subject 5

O'1-O'2	Orbitofrontal cortex R (sic)
O'6-O'7	Orbitofrontal cortex L
O'8-O'9	Orbitofrontal cortex L
O'11-O'12	Orbitofrontal cortex L
G'2-G'3	Cingulate gyrus (perigenual anterior) L
G'9-G'10	Inferior frontal gyrus L
G'11-G'12	Inferior frontal gyrus L

X'1-X'2	Superior frontal gyrus (mesial part) L
X'4-X'5	Superior frontal sulcus L
X'7-X'8	Superior frontal sulcus L
Y'1-Y'2	Orbitofrontal cortex L
Y'11-Y'12	Superior frontal gyrus (lateral part) L
Y'14-Y'15	Superior frontal gyrus (lateral part) L
D'2-D'3	Superior frontal gyrus (mesial part) L
D'5-D'6	Middle frontal gyrus L
D'7-D'8	Middle frontal gyrus L
F'1-F'2	Superior frontal gyrus (mesial part) L
F'6-F'7	Superior frontal gyrus (lateral part) L
R'4-R'5	Posterior operculum L
R'7-R'8	Posterior operculum L
S'8-S'9	Operculum L
H'1-H'2	Central cingulate gyrus L
H'6-H'7	Central operculum
H'8-H'9	Central operculum
H'10-H'11	Central operculum
E'4-E'5	Superior frontal sulcus L
E'6-E'7	Middle frontal gyrus L

Subject 6

O1-O2	Mesial orbital gyrus R
O7-O8	Lateral orbiral gyrus R
X6-X7	Middle frontal gyrus R
X15-X16	Superior frontal gyrus R
L2-L3	Mesial orbital gyrus R
H13-H14	Superior frontal gyrus (lateral part) R
O'2-O'3	Mesial orbital gyrus L
O'10-O'11	Lateral orbiral gyrus L
X'1-X'2	Middle frontal gyrus L
X'4-X'5	Middle frontal gyrus L
X'12-X'13	Superior frontal gyrus L
L'1-L'2	Mesial orbital gyrus L
L'4-L'5	Mesial orbital gyrus L
L'10-L'11	Middle frontal gyrus L
G'10-G'11	Inferior frontal gyrus L
G'14-G'15	Inferior frontal gyrus L
E'3-E'4	Superior frontal gyrus (mesial part) L
E'8-E'9	Middle frontal gyrus L
E'12-E'13	Middle frontal gyrus L
H'12-H'13	Middle frontal gyrus L (posterior)

Subject 7

C10-C11	Superior temporal sulcus R
C15-C16	Superior temporal gyrus (posterior part) R
E5-E6	Cuneus R
E12-E13	Angular gyrus R
Q1-Q2	Parietal cingulate gyrus R
Q13-Q14	Supramarginal gyrus R
Q15-Q16	Supramarginal gyrus R
L1-L2	Postcentral cingulate gyrus R
L11-L12	Inferor parietal lobule R
L13-L14	Inferor parietal lobule R
L15-L16	Inferor parietal lobule R
Y1-Y2	Central cingulate gyrus R
Y3-Y4	Central cingulate gyrus R
Y8-Y9	Precentral gyrus R
Y10-Y11	Postcentral gyrus R
Y13-Y14	Postcentral gyrus R
N1-N2	Central cinguate gyrus
N7-N8	Superior frontal sulcus R
N12-N13	Precentral gyrus R
M1-M2	Frontal mesial (SMA) R
M6-M7	Superior frontal sulcus R
M10-M11	Precentral gyrus R
H1-H2	Precentral cingulate gyrus R
J1-J2	Superior parietal lobule R
J7-J8	Superior parietal lobule R

Subject 8

B1-B2	Hippocampus R
B10-B11	Middle temporal gyrus (part. ant.) R
T2-T3	Insula (posterior part gyri brevi) R
T6-T7	Superior temporal gyrus (part. ant.) R
U3-U4	Superior temporal sulcus R
U5-U6	Superior temporal sulcus R
X8-X9	Supramarginal gyrus R
Q1-Q2	Precuneus R
Q8-Q9	Angular gyrus R
Q10-Q11	Angular gyrus R
P2-P3	Parietal cingulate gyrus R
P14-P15	Inferior parietal lobule R
M1-M2	Precentral cingulate gyrus R
M6-M7	Sulcus between sup. and med. frontl gyrus R
M8-M9	Sulcus between sup. and med. frontl gyrus R

Subject 9

X1-X2	Straight gyrus R
X7-X8	Superior frontal gyrus (lateral part) R
X10-X11	Superior frontal gyrus (lateral part) R
G1-G2	Cingulate gyrus (genu) R
G12-G13	Middle frontal gyrus R
K1-K2	Cingulate gyrus (genu) R
D11-D12	Middle frontal gyrus R
E1-E2	Superior frontal gyrus (mesial part) R
E4-E5	Lateral part of superior frontal sulcus R
E7-E8	Middle frontal gyrus R
J1-J2	Superior frontal gyrus (mesial part) R
J4-J5	Superior frontal gyrus (lateral part) R
J7-J8	Superior frontal gyrus (lateral part) R
F10-F11	Middle frontal gyrus R
N1-N2	Postcentral cingulate gyrus R
Y1-Y2	Central cingulate gyrus R
Y3-Y4	Superior frontal gyrus (mesial part) R
Y7-Y8	Superior frontal gyrus (lateral part) R
L2-L3	Sulcus postcentral R
L7-L8	Precentral gyrus R
M1-M2	Superior frontal gyrus (mesial part) R
M5-M6	Superior frontal gyrus (lateral part) R
P10-P11	Posterior parietal lobule R
P12-P13	Posterior parietal lobule R

Subject 10

G2-G3	Anterior cingulate gyrus R
G13-G14	Inferior frontal gyrus R
H1-H2	Cingulate gyrus (genu) R
H3-H4	Cingulate gyrus (genu) R
H11-H12	Inferior frontal gyrus R
R7-R8	Operculum (mesial)
R11-R12	Operculum
E1-E2	Superior frontal gyrus (mesial part) R (anterior)
E6-E7	Lateral part of superior frontal gyrus (anterior)
F1-F2	Superior frontal gyrus (mesial part) R (posterior)
F6-F7	Lateral part of superior frontal gyrus (posterior)
L9-L10	Superior frontal gyrus
J1-J2	Cingulate gyrus R
K2-K3	Superior frontal gyrus R
K4-K5	Superior frontal gyrus R
